# Colon Necrosis Due to Sodium Polystyrene Sulfonate with and without Sorbitol: An Experimental Study in Rats

**DOI:** 10.1371/journal.pone.0137636

**Published:** 2015-09-28

**Authors:** Isabelle Ayoub, Man S. Oh, Raavi Gupta, Michael McFarlane, Anna Babinska, Moro O. Salifu

**Affiliations:** 1 Department of Medicine, SUNY Downstate Medical Center, Brooklyn, New York, United States of America; 2 Department of Medicine, Ohio State University, Columbus, Ohio, United States of America; 3 Department of Pathology, SUNY Downstate Medical center, Brooklyn, New York, United States of America; Universidade Federal do Rio de Janeiro, BRAZIL

## Abstract

**Introduction:**

Based on a single rat study by Lillemoe et al, the consensus has been formed to implicate sorbitol rather than sodium polystyrene sulfonate (SPS) as the culprit for colon necrosis in humans treated with SPS and sorbitol. We tested the hypothesis that colon necrosis by sorbitol in the experiment was due to the high osmolality and volume of sorbitol rather than its chemical nature.

**Methods:**

26 rats underwent 5/6 nephrectomy. They were divided into 6 groups and given enema solutions under anesthesia (normal saline, 33% sorbitol, 33% mannitol, SPS in 33% sorbitol, SPS in normal saline, and SPS in distilled water). They were sacrificed after 48 hours of enema administration or earlier if they were very sick. The gross appearance of the colon was visually inspected, and then sliced colon tissues were examined under light microscopy.

**Results:**

1 rat from the sorbitol and 1 from the mannitol group had foci of ischemic colonic changes. The rats receiving SPS enema, in sorbitol, normal saline, distilled water, had crystal deposition with colonic necrosis and mucosal erosion. All the rats not given SPS survived until sacrificed at 48 h whereas 11 of 13 rats that received SPS in sorbitol, normal saline or distilled water died or were clearly dying and sacrificed sooner. There was no difference between sorbitol and mannitol when given without SPS.

**Conclusions:**

In a surgical uremic rat model, SPS enema given alone or with sorbitol or mannitol seemed to cause colon necrosis and high mortality rate, whereas 33% sorbitol without SPS did not.

## Introduction

Sodium polystyrene sulfonate (SPS) has long been used to treat hyperkalemia in chronic kidney disease. The first case report of colon necrosis in association with the use of SPS was presented by Arvanitakis et al in 1973 [1]. The authors of this report attributed the colon necrosis to the SPS enema, solely on the basis of the fact that all 3 patients developing colon necrosis received SPS enema. Years later, Lillemoe et al reported 5 cases of colon necrosis in kidney transplant recipients following the administration of SPS enema in a 70% sorbitol solution. Although the original case report by Arvanitakis et al did not mention the use of sorbitol along with SPS, Lillemoe et al assumed that those patients of Arvanitakis et al received SPS in sorbitol solution, because at the time SPS was given almost always in a sorbitol solution. Hence, according to their reasoning, colon necrosis could be due either to SPS or to sorbitol, and they carried out rat experiments in order to determine whether the culprit was SPS or sorbitol. The conclusion of this study appeared straightforward: Sorbitol, not SPS, was the culprit for colon necrosis; colon necrosis occurred in 100% of rats that received SPS in sorbitol or sorbitol alone, whereas none of the rats that received SPS alone developed the complication [2]. Based on the results of this rat experiment, a general consensus has been firmly established to conclude that all cases of colon necrosis in humans receiving SPS were due to sorbitol that is concomitantly administered with SPS [2]. Since this report, numerous additional case reports of colon necrosis in patients receiving SPS, mostly with sorbitol, have appeared in the literature. Many of the patients were in postoperative states at the time of the complication [3–5]. Colon necrosis also occurred in subjects who received SPS without sorbitol [6–8]. Polystyrene sulfonate is most often available as sodium salt, i.e., SPS, but some patients who developed the complication received the resin in the form of calcium polystyrene sulfonate [9–11].

Although not stated implicitly, the interpretation of the Lillemoe’s rat study is that sorbitol causes colon necrosis by its unique chemical property. We thought on the other hand that the colon damage by sorbitol, if it occurs, is due to its extreme hypertonicity and volume, not its unique chemical property. Our calculation showed that the total solute content of the sorbitol solution given to the rat by enema in Lillemoe's experiment was more than the entire solutes content of the rat. 70% sorbitol solution is very hypertonic, but because the solution is expressed in weight for weight, it contains 90 g of sorbitol in 100 ml of water. In other words, the sorbitol used in the experiment is actually a 90% solution when expressed as weight for volume. Thus, the solution has an osmolality of 12,824 mOsm/L, almost 46 times the normal plasma osmolality. It was our hypothesis that colon damage due to sorbitol solution is the result of extreme dehydration of the colonic mucosal cells by transfer of water from the mucosal cells into the colonic lumen caused by hypertonic solution, and that it was unrelated to the specific nature of the chemical. The hypothesis was further advanced to predict that any solution, such as mannitol, given in a similarly high concentration and volume would cause colon necrosis.

## Methods

The study was performed at SUNY Downstate Medical Center after obtaining the approval by its animal care and use committee. Twenty-six Sprague-Dawley male rats weighing 200 to 250 gm underwent 5/6 nephrectomy. Two weeks post-surgery, blood was drawn from all rats to confirm, by measurements of BUN and serum creatinine, the presence of uremia. The rats were divided into 6 groups, and were given rectal enemas with the solutions of the following compositions; **Group I**: 3 rats (control), 5 mL of normal saline, **Group II**: 5 rats, 5 mL of 33% sorbitol solution, **Group III**: 5 rats, 5 mL of 33% mannitol solution, **Group IV**: 5 rats, 5ml of SPS dissolved in a 33% sorbitol solution, **Group V**: 3 rats, 5ml of SPS dissolved in normal saline, **Group VI**: 5 rats, 5 ml of SPS dissolved in distilled water. There were only 3 rats each in Group I and V, whereas there were 5 rats in the other groups. We decided to use these 6 rats that received neither sorbitol nor mannitol to serve as control against rats receiving sorbitol or mannitol, since our original hypothesis was both hypertonic sorbitol and mannitol would be toxic. Of course our prediction was proven wrong later.

The day before the enema, each rat was provided with 2 pallets of renal diet. Enema solution was administered under anesthesia with 1–5% isoflurane mixed with 100% oxygen via nose cone. The condition of the rats was monitored twice daily after enema. Pain or distress was assessed on the basis of decreased activity, abnormal postures, poor grooming, change in body temperature, self-aggregation behavior, changes in pulse or respiratory rate and abnormal physical responses to touch. During the entire study period, Buprenorphine was available for analgesia for the post procedure administration, but there was no need to use the medication on the basis of the above pain assessment. Rats underwent euthanasia 48 hours after enema using carbon dioxide; Anesthesia with isoflurane preceded euthanasia in all cases. The entire colon was removed; the gross appearance of the colon was visually inspected, and then tissue slices of representative areas were examined under light microscopy. Very sick rats were euthanized as per the experimental protocol. Hence, some rats in groups IV-VI underwent euthanasia earlier than 48 hours. Rats were judged to be very sick when they were sluggish, in a hunched posture with difficulty moving or breathing. Further details are presented in the supporting information (S1 Text).

### Statistical Analysis

Gross pathologic changes consistent with colonic necrosis were described in detail and chi square analysis was used to compare the groups for statistical significance as appropriate. A p value <0.05 was considered significant.

## Results

**Fig 1:**
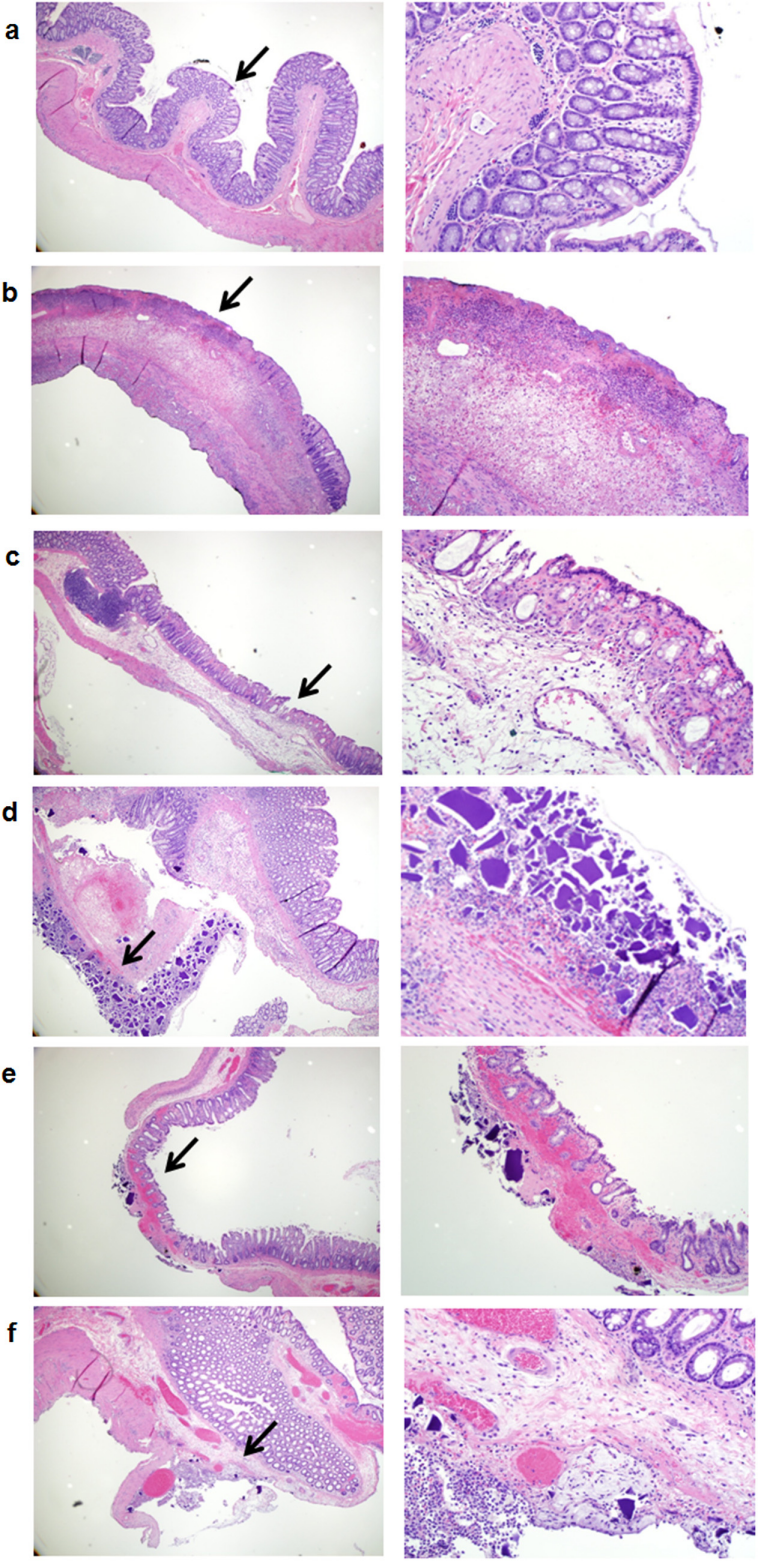
Pathologic findings of rats’ colon with the different enema solutions. Fig 1a: Control rats with distal colon showing preserved crypt architecture and minimal inflammation. Fig 1b: Sorbitol (33%) with transmural necrosis showing trans-mural necrosis. Fig 1c: Mannitol (33%) with transmural ischemia. Fig 1d: SPS with sorbitol (33%) showing transmural infarction with crystal deposition. Fig 1e: SPS with normal saline with trans-mural ischemia and crystal deposition. Fig 1f: SPS with water showing sub-mucosal edema and crystal deposition in the serosa with accompanying acute inflammation. Hematoxylin-Eosin of cross-section of rat colons: images on the left panel (Fig 1a-f) represent original magnifications x40; images on the right panel (Fig 1a-f) represent original magnifications x200. Black arrows indicate magnified area.

No pathologic changes were seen in group I (control rats) (Fig 1A). One rat in each of the group II (rats which received 33% of sorbitol) and group III (rats which received 33% mannitol) showed foci of transmural ischemic changes in the distal colon consistent with surface ulceration, submucosal edema, transmural acute inflammation and necrosis (Fig 1B and 1C). All rats in group I, II and III survived until sacrificed. In group IV, V and VI (rats which received SPS in 33% sorbitol, SPS in normal saline, and SPS in distilled water respectively) the pathologic changes showed SPS crystal deposition mostly in the serosa accompanied by acute inflammation, necrosis and mucosal erosion. The crystal deposition was focal and the accompanying inflammation extended laterally and transmurally to involve the mucosa. Separate areas of mucosal damage were present. These changes were seen mainly in the distal colon. However, two rats in group IV (SPS alone) and one rat in group V (SPS and sorbitol) showed damage in the entire colon. In group IV, 4 rats showed the crystal deposition and 2 had both transmural ischemia and the crystal deposition (Fig 1D). In group V all 3 rats had the crystal deposition and one of the rat colon showed extensive mucosal ulceration (Fig 1E). In group VI (SPS and water), 4 rats showed the crystal deposition and 2 had mucosal ulcerations (Fig 1F)

Unlike groups I, II and III rats all of which survived until the end of the experiment, 11 of 13 rats in groups IV, V, and VI either died within 48h or were sacrificed before the end of the experiment. In group IV, 3 rats were found dead (2 with stiff body on palpation and 1 with soft body on palpation) within 24 h post enema with an undetermined duration between death and colon extraction and 2 rats were sacrificed before the end of experiment because they were very sick. In group V, one of these rats was found dead and stiff, within 24 h post enema with an undetermined duration between death and colon extraction, and 1 rat was very sick and dying, and was sacrificed before the end of procedure. One rat survived until 48 hours despite colon necrosis. In group VI, one rat survived until the end of the experiment, and 4 of 5 rats were very sick and dying within 24 h post enema, and were sacrificed before the end of the experiment.

The results on death or survival in those rats that received sorbitol or mannitol and those that did not receive either solution are shown in Table 1. 15 rats received either solution, and of these 5 died and 10 survived. 11 rats did not receive either solution, and of these 6 died and 5 survived. The sum of chi square values comparing the two groups was 3.937 and p = 0.07.

**Table 1:** Death versus survival depending on the exposure to sorbitol or mannitol.

	Sorbitol or Mannitol	No Sorbitol or Mannitol
Died	5	6
Survived	10	5

The results on death or survival after grouping of rats into those that received SPS and those that did not are shown in Table 2. 13 rats received SPS and 13 did not. Of 13 that received SPS, 11 died and 2 survived. All the 13 rats that did not receive SPS survived. The difference between the two groups was considered highly significant, as the sum of chi square values comparing the two groups was 19.07 and p = 0.00007.

**Table 2:** Death versus survival depending on exposure to SPS.

	SPS	No SPS
Died	11	0
Survived	2	13

## Discussion

Colon necrosis is not a rare complication in patients treated with SPS used mostly with sorbitol solution. The current prevailing opinion that puts the blame on sorbitol solution is based on a single rat study by Lillemoe et al [2]. Contrary to our original hypothesis that extreme hypertonicity of sorbitol solution is the mechanism of colon necrosis, sorbitol solution does not appear to contribute to colon necrosis. Unexpectedly, our study showed that SPS, not sorbitol, is the main culprit for colon necrosis.

Our hypothesis was based on the assumption that the colon has sufficient water permeability as it is known to possess various aquaporins [12], and the extremely high colonic osmolality by sorbitol or mannitol solution would severely dehydrate colonic mucosal cells resulting in necrosis. The negative results indicate that the colon has limited water permeability. Expression of aquaporin 4 in the colon has been shown, but apparently it is expressed only on the basolateral membrane [13], and therefore may not participate in the trans-cellular water transport between the lumen and the mucosal cell. Similarly, the colon is not permeable to carbon dioxide, indicating the absence of expression of aquaporin 1 on the luminal membrane [14]. Tap water enema is often used clinically, and this also indicates that colon has limited water permeability.

As shown in our study, the culprit for colon necrosis appears to be SPS per se, not sorbitol. The mechanism of colon damage by SPS is not known, but according to a study, in 9 of 11 patients who received SPS in sorbitol solution, the endoscopic examination of the upper GI tract showed kayexalate crystals adhered to the damaged mucosa of the esophagus, stomach and the duodenum, suggestive of the damaging effect of SPS [15]. The observation in our study that there was more premature death (3 of 5 rats receiving SPS with sorbitol) suggests a possibility that the combination increase the toxic effect of the SPS crystals. However the number of observations is too small to draw a firm conclusion on this point.

The only clinical study that has been designed to explore the question on the causality of SPS and/or sorbitol use for colon necrosis in humans is a retrospective cohort study. The percentage of colon necrosis was 0.14% among in-patients (3/2194) and 0.07% (79/121,197) in the control group, a difference that was not statistically significant. None of the outpatients exposed to SPS developed colon necrosis. This suggests that severe illness per se predisposes to colon necrosis. The likelihood that other risk factors, such as renal failure, anesthesia induced ileus, inflammatory and immunosuppressive states, influences the development of colon necrosis is suggested by the very high incidence of colonic complications in patients post renal transplantation surgery [8,16,17].

Our results are different from Lillemoe’s findings. One plausible explanation for the greater toxicity of sorbitol solution in Lillemoe’s rat experiment is the use of a much higher hypertonic solution than in the present work. The solution of 70% sorbitol is 90% when expressed as weight for volume. The current sorbitol solution used clinically and also in our experiment is 33%, and is expressed as weight for volume. However, the actual osmolality difference between the two is far greater than expected from the difference of 90% versus 33%, because osmolality depends on solute concentration per given water volume. For the same volume, a 90% sorbitol solution contains much less water than a 33% solution, and consequently osmolality of 90% sorbitol solution is more than 5 times that of 33% solution. However, the osmolality of both sorbitol and mannitol solutions used in our experiment is 2376 mOsm/L, which is 8.5 times normal plasma osmolality, sufficiently high to cause severe mucosal damage by dehydration, if the colon had sufficient water permeability. If the colon had limited water permeability, 70% solution may cause colon damage, but not 33% solution. Other possible explanations are differences in the experimental protocols between the two. Our rats had 5/6 nephrectomy 2 weeks before the experiments, and Lillemoe's rats underwent bilateral total nephrectomy on the day of the experiment. They administered enema on post-surgery days 0 and 1; we administered enema 2 weeks post-surgery.

Our study had some limitations. These findings may not be extrapolated to the non-surgical, non-anesthetized CKD humans. Furthermore, the volume of SPS solutions we used in our rat experiment was proportionately much greater than that used clinically in the treatment of hyperkalemia. 5ml volume is 1/20 of 100ml, a typical volume used clinically, but a typical rat weight is 1/300 to 1/400 of a typical human weight. We weren’t able to determine the duration between death and extraction of colon when death occurred prematurely (within 24h post enema injection). Finally the lack of aquaporins in the colon for transmembrane water movement may protect the colon upon the use of hypertonic sorbitol, but sorbitol may cause damage to the intestine when the solution is administered orally, since the small intestine clearly has ample expression of aquaporins.

In summary, the data from our experiments on a surgical uremic rat model suggest that SPS, not sorbitol, is the main culprit for colon necrosis, and the extreme hypertonicity of sorbitol solution does not appear to contribute to colon necrosis probably because of the limited water permeability of the colon.

## Supporting Information

S1 Text Details of the experiment (DOCX)

## Abbreviations

SPS: Sodium polystyrene sulfonate
CKD: Chronic Kidney Disease
