# Supplementary material for: Colon Necrosis Due to Sodium Polystyrene Sulfonate with and without Sorbitol: An Experimental Study in Rats
Source: PLoS One. 2015 Sep 28;10(9):e0137636. doi: 10.1371/journal.pone.0137636 (PMC4587365; doi:10.1371/journal.pone.0137636)
Supplement: S1 Text — (DOCX) [file pone.0137636.s001.docx]

Supporting Information

S 1 text: Details of the experiment

1.**Title:**

Page 1 of the manuscript

2. **Introduction** :

Page 4 and 5 in the introduction section of the manuscript

Backgroud: First paragraph

Objective: Second paragraph

3. **Methods:**

Page 6 in the method section of the manuscript

Ethical Statement: first paragraph

Study design : first and second paragraph

Experimental procedures:

# The experimental study was performed at SUNY Downstate Medical Center after obtaining the approval by the animal care and use committee. Twenty-six Sprague-Dawley male rats weighing 200 to 250 gm with5/6 nephrectomy were ordered from Charles River Laboratories International, Inc. Kingston, NY.

Two weeks post-surgery, blood was drawn from all rats to confirm, by measurements of BUN and serum creatinine, the presence of uremia.

The day before the enema, each rat was provided with 2 pallets of renal diet.

Twenty six rats were divided into 6 groups, and under anesthesia (with 1-5% isoflurane mixed with 100% oxygen via nose cone) were given rectal enemas with the solutions of the following compositions; Group I: 3 rats (control), 5 mL of normal saline, Group II: 5 rats, 5 mL of 33% sorbitol solution, Group III: 5 rats, 5 mL of 33% mannitol solution, Group IV: 5 rats, 5ml of SPS dissolved in a 33% sorbitol solution, Group V: 3 rats, 5ml of SPS dissolved in normal saline, Group VI: 5 rats, 5 ml of SPS dissolved in distilled water.

Rectal enemas were performed in a special surgical room with equipment for anesthesia.

Enema was administered between 9 - 11:30 am, under anesthesia with isoflurane inhalation (1-5% isoflurane mixed with oxygen). Anesthetic recovery included monitoring until the animals were able to maintain normal physiology. After enema administration animals were monitored twice daily, they did not require sedation and analgesia. Buprenorphine was available for analgesia. 48h after enema injection euthanasia was performed for the purpose of obtaining tissue required for this study. We understand that rats that were very sick were euthanized as per the experimental protocol. Rats were judged to be very sick when they were sluggish, in a hunched posture with difficulty moving or breathing.

Carbon dioxide has been used to euthanatize rats. It was conducted in accordance with 2013 Report of the American Veterinary Medical Association Panel on Euthanasia. Anesthesia will precede euthanasia in all cases. This was followed by an exam to confirm the absence of a heartbeat, which is a reliable indicator of death.

Then the entire colon was removed; the gross appearance of the colon was noted and representative areas were examined under light microscopy.

Experimental animals:

| Strain Name: Sprague dawley rats |
| --- |
| Sex, age/weight: male; 8 weeks old/; between 200-250g |
|  |

Source: The Charles River Laboratories International, Inc. Kingston, NY.

The animals were not genetically modified.

Housing and husbandry

Thirteen standard rat cages, top with water bottle and food pellet, with bottom water-soak-cardboard have been housed in special pathogen free room, with controlled temperature, humidity and adequate ventilation at SUNY/Downstate animal facilities.

Two rats have been housed in each cage with permanent access to food and water.

Non-contaminated and nutritionally adequate food pellet have been placed on the top of each cage daily.

Sample size :

26 rats

We assume that high concentration groups will have colonic necrosis whereas low concentration will not with an effect size of 1.

To achieve a p value of 0.05 and a power of 80% a total number of 8 rats will be required in each group, using Chi Square analysis.

Because the rats will be sacrificed in Day 3, we assume a negligible attrition rate and 8 rats is sufficient in each group.

High concentration group: 15 rats (group II,III and IV)

Low concentration groups: 8 rats (group V and VI)

And we added 3 control rats that received normal saline only

Allocating animals:

Random allocation

The rats were divided into 6 groups, and were given rectal enemas with the solutions of the following compositions; Group I: 3 rats (control), 5 mL of normal saline, Group II: 5 rats, 5 mL of 33% sorbitol solution, Group III: 5 rats, 5 mL of 33% mannitol solution, Group IV: 5 rats, 5ml of SPS dissolved in a 33% sorbitol solution, Group V: 3 rats, 5ml of SPS dissolved in normal saline, Group VI: 5 rats, 5 ml of SPS dissolved in distilled water.

Experimental outcomes:

Page 5 manuscript end of introduction section

colon necrosis in the rats as a result of high osmolality and content of the sorbitol solution, unrelated to the specific nature of the chemical

Statistical methods

Page 6 statistic section

4. **Results**

Page 7 to 9 in the result section of the manuscript

5. **Discussion**

Page 10 to 12 in the discussion section of the manuscript
